# Decoding incretin resistance: a mechanistic framework to reclassify therapeutic variability for GLP-1 receptor agonist therapy

**DOI:** 10.1007/s00726-025-03488-9

**Published:** 2025-11-14

**Authors:** Almir Fajkić, Yun Wah Lam, Andrej Belančić

**Affiliations:** 1 Department of Pathophysiology, Faculty of Medicine, University of Sarajevo, Sarajevo, 71000 Bosnia and Herzegovina; 2 Department of Health Sciences, School of Nursing and Health Sciences, Hong Kong Metropolitan University, Hong Kong SAR, China; 3 Department of Basic and Clinical Pharmacology and Toxicology, Faculty of Medicine, University of Rijeka, Rijeka, Croatia

**Keywords:** Incretin resistance, GLP-1 receptor agonist, Type 2 diabetes mellitus, Therapeutic variability, Mechanistic classification, Personalized medicine

## Abstract

This article introduces a mechanistic framework to reclassify suboptimal responses to GLP-1 receptor agonists. It defines three mechanistic subtypes of incretin resistance—receptor-level, post-receptor, and secretory—highlighting their distinct pathways and therapeutic implications. This model promotes personalized care by moving beyond the oversimplified ‘non-responder’ classification.

GLP-1RAs have transformed the treatment of type 2 diabetes mellitus (T2DM), providing not only potent glycemic control but also weight loss and cardiorenometabolic protection (Psaltis et al. 2025). Despite their growing clinical applications, a considerable proportion of patients fail to achieve meaningful glycemic or weight loss targets and are broadly categorized as ‘non-responders’ (Squire et al. 2025). Such all-or-nothing labeling, though convenient, does not necessarily reflect the biological complexity underlying incretin functions. In this context, we propose a Mechanistic Incretin Resistance (MIR) model that offers a structured framework for interpreting the spectrum of responsiveness to incretin-based therapies, shifting the focus from binary classification of clinical outcomes to a more nuanced understanding of therapeutic heterogeneity.

The concept of incretin resistance is not novel. As early as 2011, reports have shown a blunted insulinotropic response to GLP-1 and glucose-dependent insulinotropic polypeptide (GIP) in some individuals with T2DM, despite normal or even elevated circulatory levels of these hormones (Nauck 2011). However, unlike insulin resistance, which is routinely stratified into hepatic, muscular, and adipose-specific phenotypes, incretin resistance is not widely adopted in clinical classification. This omission is particularly conspicuous given the widespread enthusiasm surrounding incretin-based therapies and long-standing evidence of heterogeneous patient responsiveness. Yet, no formal classification system exists to account for these divergent outcomes or guide treatment decisions.

We argue that the overarching phrase “therapeutic failure” obscures specific mechanistic subtypes of incretin resistance, each with different diagnostic and therapeutic implications. To address this, we propose a mechanistic framework comprising three subtypes of incretin resistance—receptor-level defects, post-receptor signaling abnormalities, and secretory deficiencies—without yet prescribing diagnostic thresholds or clinical cut-offs, which remain to be defined in future studies (Table 1).

**Table 1:** Mechanistic framework of incretin resistance in GLP-1RA therapy: Mechanisms, clinical Features, and therapeutic implications

Mechanistic subtype	Underlying mechanism	Clinical indicators	Therapeutic implications
Receptor-level resistance	Downregulation, desensitization, or internalization of GLP-1R/GIPR at target tissues, particularly β-cells.	Blunted glycaemic or weight response to high-dose GLP-1RA; minimal gastrointestinal side effects; long T2DM duration.	Consider dual agonists (e.g. tirzepatide) to engage compensatory pathways; GLP-1RA dose escalation often futile.
Post-receptor resistance	Impaired intracellular transduction (cAMP/PKA/PI3K pathways), β-arrestin-2 depletion, ER stress, or G-protein decoupling.	Inadequate response despite preserved receptor function; poor efficacy with both GLP-1RA and DPP-4i.	Current therapies largely ineffective; experimental strategies (e.g. biased agonists, allosteric modulators) under development.
Secretory deficiency	Impaired endogenous incretin (GLP-1/GIP) release due to enteroendocrine cell dysfunction.	Rapid clinical improvement with low-dose GLP-1RA or DPP-4i; pronounced GI symptoms at low doses.	Excellent responders to exogenous incretin therapy; titrate cautiously to mitigate gastrointestinal intolerance.

*GLP-1RA* Glucagon-like peptide-1 receptor agonist, *GIPR* Glucose-dependent insulinotropic polypeptide receptor, *DPP-4i* Dipeptidyl peptidase-4 inhibitor, *ER* Endoplasmic reticulum, *T2DM* Type 2 diabetes mellitus

The first type is receptor-level resistance, characterized by the reduced expression or faulty functionality of the GIPR or GLP-1R at the target tissue level, particularly in β-cells of the pancreas. This mechanism is well-documented in T2DM and may result from chronic hyperglycemia, inflammation, or epigenetic modifications affecting receptor gene transcription (Rajan et al. 2015). Sustained hyperglycemia has been shown to downregulate GLP-1R and GIPR expression in pancreatic islets and promotes internalization of GLP-1R via PKA-dependent mechanisms, impairing insulinotropic signaling (Rajan et al. 2015). Beyond β-cells, reduced GLP-1R expression has also been reported in extrapancreatic tissues, suggesting broader physiological consequences (Grunddal et al. 2022). In addition to decreased expression, defective receptor trafficking and desensitization dynamics further compromise functionality, particularly in the case of GIPR (Manchanda et al. 2023). Clinically, this type is characterized by a poor glycemic or weight response with increasing doses of GLP-1RAs. In patients with putative receptor-level resistance, tirzepatide should be framed as a pragmatic rather than definitive option. Current data indicate that its metabolic efficacy is largely GLP-1R–dependent (Fisman and Tenenbaum 2021). In contrast, GIPR agonism appears to mitigate GLP-1RA-induced gastrointestinal intolerance, facilitating titration to higher therapeutic exposure rather than directly restoring β-cell responsiveness (Guccio et al. 2022). This may explain why clinical benefit persists despite partial GIPR dysfunction, but it does not imply mechanistic reversal of resistance. Beyond tirzepatide, GLP-1/glucagon co-agonists (e.g., cotadutide) and triple agonists (e.g., retatrutide) introduce hepatic and energy-expenditure pathways independent of classical incretin signaling. These agents offer a mechanistically distinct route around β-cell receptor impairment and merit prospective, mechanistic type-stratified evaluation before any firm clinical positioning (Yan et al. 2025).

The second is post-receptor resistance with normally functioning GLP-1R or GIPR receptors but dysfunctional downstream intracellular signaling, most pronounced within the cAMP/PKA and PI3K/Akt cascades. Both receptors are G-protein-coupled receptors (GPCRs) that activate adenylate cyclase, leading to increased intracellular cAMP levels and PKA-mediated stimulation of insulin secretion in pancreatic β-cells (Capozzi et al. 2025; Shilleh et al. 2024). Nevertheless, with diabetogenic insults such as chronic hyperglycemia and lipotoxicity, the pathway succumbs to dysregulation despite normal receptor expression. For GLP-1R, the production of cAMP and activation of PKA are reduced in hyperglycemia, accompanied by induced receptor internalization, thereby impairing its insulinotropic action. GIPR signaling is inhibited at the PI3Kγ level with critical actin remodeling and secretory functions of insulin. In addition, modulators such as β-arrestin 2 are downregulated in diabetes, further compromising signal transduction. Endoplasmic reticulum stress may also transform the coupling of the G protein of GLP-1R to Gq instead of Gs (Zaïmia et al. 2023). Currently, no approved therapy directly targets these intracellular defects. Experimental agents, such as biased agonists and positive allosteric modulators, are promising strategies but are currently in preclinical or preliminary clinical trials (Malik and Li 2022).

The third type, secretory resistance, is characterized by a reduced endogenous secretion of GLP-1 and/or GIP in response to nutrient ingestion. Incretin hormones are typically released by intestinal K-cells (GIP) and L-cells (GLP-1) following exposure to carbohydrates, lipids, and proteins, and collectively contribute to the majority of postprandial insulin secretion in healthy individuals, with GIP accounting for up to 60–80% of the incretin effect. In T2DM, a blunted GLP-1 release has been variably observed, likely due to chronic hyperglycemia, insulin resistance, and desensitization of enteroendocrine cells. This impairment limits the physiological amplification of postprandial insulin release, contributing to hyperglycemia (Wolfe et al. 2025). Unlike the other two types, receptor function and intracellular signaling remain largely intact in secretory resistance, making these individuals particularly amenable to exogenous incretin-based therapies. GLP-1 receptor agonists compensate for hormonal deficiencies and are effective regardless of endogenous hormone levels. Dual agonists, such as tirzepatide, activate both GLP-1 and GIP pathways and may offer additive benefits. Moreover, a favorable response to DPP-4 inhibitors, which prolong the action of endogenously secreted incretins, may serve as a surrogate marker of preserved receptor functionality in the context of impaired secretion (Bailey and Flatt 2024) (Fig. 1).

**Fig. 1:**
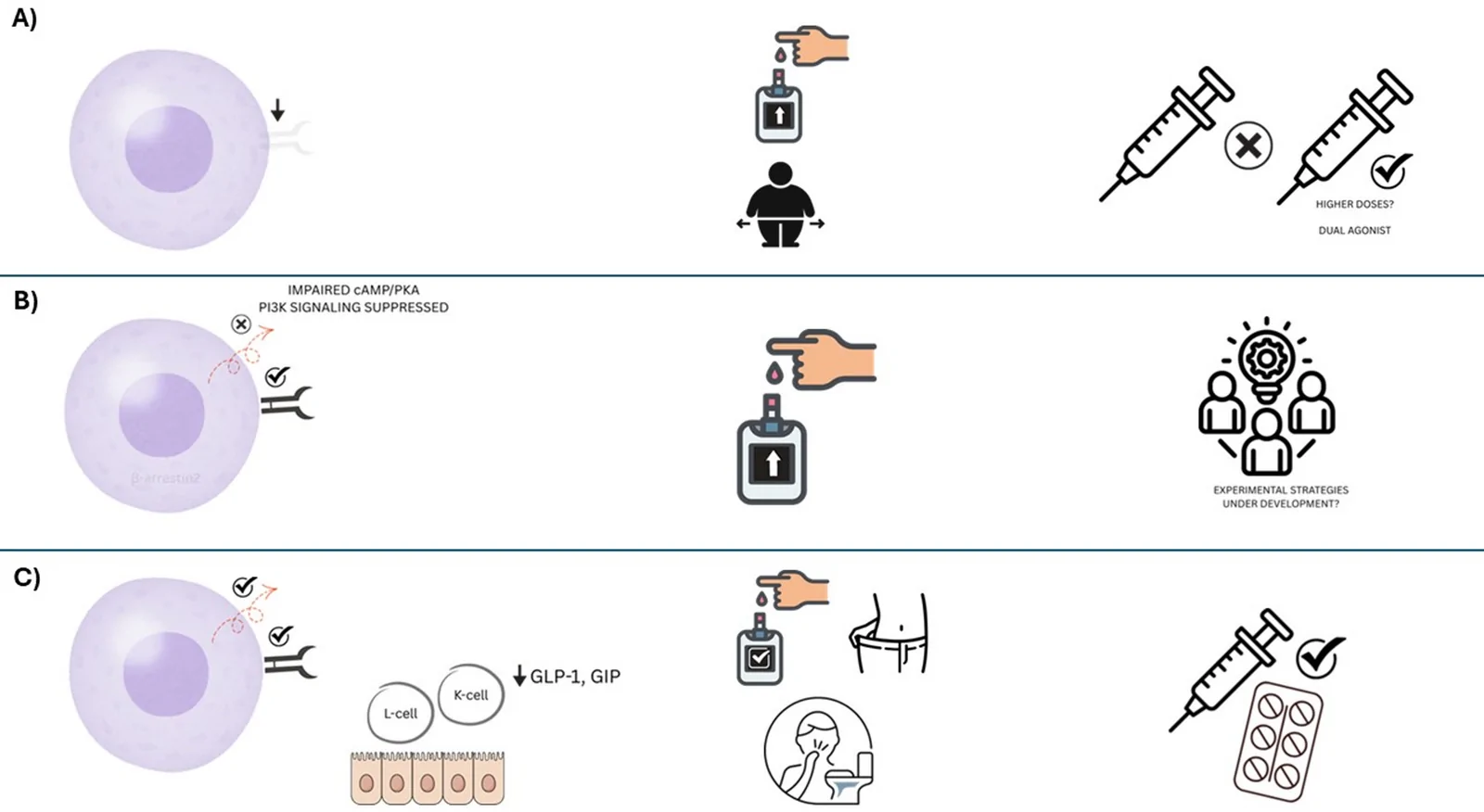
A mechanistic framework for incretin resistance in GLP-1RA therapy

MIR typing is not yet readily translatable to clinical practice because of the current lack of properly validated diagnostic tools. Nevertheless, we contend that therapeutic response itself can serve as a pragmatic surrogate that informs the assignment of type. For example, failure to achieve a significant glycemic response following a proper trial of GLP-1RA therapy, despite demonstrated adherence and lifestyle optimization, may signify receptor-level or post-receptor resistance. Conversely, a robust response to low-dose GLP-1RA or DPP-4 inhibitor may suggest a secretory type, characterized by impaired endogenous hormone release with normal downstream pathways.

This model also provides new avenues for reinterpreting the gastrointestinal side effects of GLP-1RAs. It is conceivable that secretory type patients with relative receptor hypersensitivity due to lifelong deficiency of the natural hormone may experience more pronounced gastrointestinal symptoms, such as nausea, even at low or subtherapeutic doses. In contrast, receptor-resistant patients may tolerate higher doses with analogous adverse symptoms but derive less therapeutic benefit. Clarification of such heterogeneity is possible to permit more individualized titration of doses and achieve greater compliance through balancing tolerability and efficacy.

The MIR concept can also guide the sequencing of therapies following GLP-1RA failure. In current practice, patients who do not respond to GLP-1RA therapy are typically transitioned to basal insulin or SGLT2 inhibitors, typically without reference to underlying incretin-resistance mechanisms. In contrast, a mechanistic type-guided approach allows a more rational clinical decisions: patients with receptor-level resistance may switch to tirzepatide therapy, those with secretory types may require conservative dose titration to minimize side effects, while individuals with post-receptor resistance may need alternative treatments as they are unlike to benefit from incretin-based therapies. These options might be speculative at this stage but they are consistent with broader trends in personalized care and the optimal use of resources.

From a research perspective, MIR typing offers a valuable framework for designing more targeted clinical trials that evaluate differential therapeutic responses across mechanistically defined subgroups. Instead of post hoc responder analyses in current research, future studies could involve mechanistic type-informed stratification at the point of enrollment to achieve both maximal statistical power and clinical relevance. Further, biomarker identification research, based on receptor expression profiling, GLP-1R polymorphisms, or downstream signaling molecules, may permit precision matching of the patient to incretin-based therapy. To support this approach, a complementary clinical model can be used to integrate measurements of early response to therapy with clinical and biochemical indicators, including fasting, postprandial C-peptide levels, and clinical data such as diabetes duration, BMI, and hepatic steatosis. Such instruments maybe indirect but they can give us actionable daily information that can help fine-tuned patient subtyping and therapeutic decisions. Ultimately, this PIR-centric model could pave the way towards the development for more personalized, mechanism-based treatment strategies in metabolic diseases.

We are not advocating MIR as a rigid classification into types, but rather as a heuristic framework that encourages clinicians to consider pathophysiological heterogeneity before labeling an individual as a “non-responder.” MIR is intended as an exploratory model to trigger debate, generate hypotheses, and inform mechanism-based clinical trials. While mechanistic type boundaries may be blur, and intermediate mechanistic types are likely, the clinical relevance of the model lies in its ability to reframe therapeutic inertia not as a failure of the drug itself, but as a failure to recognize and address the biological heterogeneity of response. The proposed mechanistic typic model interlaces current pathophysiological understanding with therapeutic utility to frame a conceptual bridge between bench science and clinical decision-making at the bedside. Verification in clinical research is necessary in the long run. Still, the adoption of an MIR framework may already provide clinicians with a more nuanced perspective through which to understand and respond to therapeutic failure within incretin-based therapeutic paradigms.

## Implementation and real-world barriers

The transition from a conceptual, mechanism-informed framework to actual clinical deployment requires confronting several barriers. First, mechanistic type assignment must rely on simple, low-cost signals, such as early HbA1c and weight changes at 4–6 weeks, prior response to DPP-4 inhibitors, diabetes duration, and the presence of gastrointestinal adverse events, to avoid dependence on high-cost biomarkers or imaging. Second, workflow integration is critical: any algorithm demanding frequent laboratory testing or specialized software risks rapid abandonment in routine practice. Third, cost-effectiveness and clinical inertia remain major obstacles; history shows that complex predictive tools, even when accurate, rarely survive if they slow decision-making or lack reimbursement pathways. To balance these realities, we outline a two-tier approach: a pragmatic, clinic-ready tier using easily accessible parameters for preliminary stratification, and a research-intensive tier (with C-peptide dynamics, CGM-derived postprandial profiles, and exploratory biomarkers), reserved for prospective studies. This design favors feasibility over perfection, positioning the framework as a heuristic for hypothesis generation rather than an immediate prescriptive algorithm.

## Data Availability

No new data was generated. Available upon reasonable request sent to the corresponding author.
